# Complete genome sequencing and investigation on the fiber-degrading potential of *Bacillus amyloliquefaciens* strain TL106 from the tibetan pig

**DOI:** 10.1186/s12866-022-02599-7

**Published:** 2022-07-29

**Authors:** Zhenda Shang, Suozhu Liu, Yanzhen Duan, Chengling Bao, Jian Wang, Bing Dong, Yunhe Cao

**Affiliations:** 1 State Key Laboratory of Animal Nutrition, College of Animal Science and Technology, China Agricultural University, 100193 Beijing, People’s Republic of China; 2 College of Animal Science, Tibet Agricultural and Animal Husbandry University, 860000 Nyingchi, People’s Republic of China

**Keywords:** *Bacillus amyloliquefaciens*, Tibetan pigs, Complete genome sequence, Cellulose degradation characteristics, Enzymatic activity

## Abstract

**Background:**

Cellulolytic microorganisms are considered a key player in the degradation of feed fiber. These microorganisms can be isolated from various resources, such as animal gut, plant surfaces, soil and oceans. A new strain of *Bacillus amyloliquefaciens*, TL106, was isolated from faeces of a healthy Tibetan pigs. This strain can produce cellulase and shows strong antimicrobial activity in mice. Thus, in this study, to better understand the strain of *B. amyloliquefaciens* TL106 on degradation of cellulose, the genome of the strain TL106 was completely sequenced and analyzed. In addition, we also explored the cellulose degradation ability of strain TL106 in vitro.

**Results:**

TL106 was completely sequenced with the third generation high-throughput DNA sequencing. In vitro analysis with enzymatic hydrolysis identified the activity of cellulose degradation. TL106 consisted of one circular chromosome with 3,980,960 bp and one plasmid with 16,916 bp, the genome total length was 3.99 Mb and total of 4,130 genes were predicted. Several genes of cellulases and hemicellulase were blasted in Genbank, including β-glucosidase, endoglucanase, ß-glucanase and xylanase genes. Additionally, the activities of amylase (20.25 U/mL), cellulase (20.86 U/mL), xylanase (39.71 U/mL) and β-glucanase (36.13 U/mL) in the fermentation supernatant of strain TL106 were higher. In the study of degradation characteristics, we found that strain TL106 had a better degradation effect on crude fiber, neutral detergent fiber, acid detergent fiber, starch, arabinoxylan and β-glucan of wheat and highland barley .

**Conclusions:**

The genome of *B. amyloliquefaciens* TL106 contained several genes of cellulases and hemicellulases, can produce carbohydrate-active enzymes, amylase, cellulase, xylanase and β-glucanase. The supernatant of fermented had activities of strain TL106. It could degrade the fiber fraction and non-starch polysaccharides (arabinoxylans and β-glucan) of wheat and highland barley. The present study demonstrated that the degradation activity of TL106 to crude fiber which can potentially be applied as a feed additive to potentiate the digestion of plant feed by monogastric animals.

**Supplementary Information:**

The online version contains supplementary material available at 10.1186/s12866-022-02599-7.

## Background

Tibetan pigs are the characteristic breed of the Qinghai Tibet plateau and graze throughout the year feeding mainly on grass [1]. Under long-term exposure to harsh natural environment conditions, Tibetan pigs have evolved to herbivorous [2], cold resistant [3], disease resistant [4], and high-qualified pork [5]. These characteristics of Tibetan pigs have attracted researchers. Possibly, these valuable characteristics of Tibetan pigs are related to the special microflora of the pigs’ intestinal tract [6]. Yang et al. [7] isolated a strain of *Bacillus subtilis* BY-2 from intestinal tracts of Tibetan pigs which produced cellulase to degrade wheat straw.

Cellulose is the most abundant and widely distributed renewable biological resource on earth. Its utilization and biotransformation are effective ways to alleviate global energy problems [8]. Consequently, processes that degrade cellulose have received much attention. Degradation of cellulose polymers are involved complementary and coordinated synergy of several enzymes (CAZymes) by performing oxidative, hydrolytic and non-hydrolytic reactions between substrates and enzymes [9]. Among micro-organisms, fungi such as *Aspergillus* and *Penicillium* that can produce cellulose degrading enzymes have been studied extensively [10, 11]. However, fungi are not suitable for large-scale production because they are sensitive to surroundings and temperatures [12]. Compared with fungi, bacteria can grow fasten and have wide ranges of desirable cultivation temperature, and pH. Bacteria can express multi-enzyme complexes, and derived from a variety of sources [13]. These characteristics of bacteria have made them the focus of research on cellulase production. Many bacterial genera can produce cellulose degrading enzymes, such as *Bacillus*, *Paenibacillus* and *Cellulomonas* [14–16].

*Bacillus amyloliquefaciens* belongs to *Bacillus* genus, which is highly related to *Bacillus subtilis* and widely distributed in nature [17]. For instance, *B. amyloliquefaciens* is present in compost, soil, plant surfaces, animal body, ensiled corn stalks, marine red tide, and mud sewage holding ponds [18, 19]. Different species of *B. amyloliquefaciens* are classified according to different identification methods and biochemical characteristics. *B. amyloliquefaciens* can synthesize a variety of cellulase enzymes, especially carboxymethyl cellulase and filter paper enzyme [20], which has potential to promote decomposition and utilization of cellulosic resources at a scale of industrial production [21]. Lee et al. [22] isolated *B. amyloliquefaciens* DL-3 from soil which produced cellulase to hydrolyze carboxymethylcellulose, cellobiose, ß-glucan and xylan. Li et al. [23] showed that *B. amyloliquefaciens* MN-8 isolated from fresh cattle manure, could produce cellulase and hemicellulase, to degrade cellulose in corn stalk. Therefore, *B. amyloliquefaciens* has the potential to improve the utilization of cellulose for animal production.

In a previous study, we isolated a strain of *B. amyloliquefaciens*, TL106, from Tibetan pig feces and demonstrated that oral administration of TL106 in mice alleviated the deterioration of intestines challenged enterohaemorrhagic *Escherichia coli in vivo* [24]. In this study, a whole genome sequencing of TL106 was carried out and its activities on cellulose degradation was characterized in vitro.

## Methods

### Ethics statement

Tibetan pigs are raised at Tibetan Pig Collaborative Research Center of Tibet Agriculture and Animal Husbandry University. This study was approved and instructed by the Tibetan Pig Collaborative Research Center of Tibet Agricultural and Animal Husbandry University, Tibet, China (unified social credit code: 12540000MB0P013721).

### DNA extraction

Strain TL106 was isolated from Tibetan pig faeces (Nyingchi, Tibet) and stored in China General Microbiological Culture Collection Center (No. CGMCC 20,391). TL106 was cultured in LB medium (5% yeast extract, 10% tryptone, 10% NaCl) at 30℃ for 24 h. Total DNA of the bacterium was extracted using QIAamp Fast DNA Stool Mini Kit (Qiagen, Hilden, Germany) according to manufacturer’s instructions. Purity and integrity of total DNA were determined using 1.2% agarose gel electrophoresis.

### Genome sequencing and assembly

Genomic DNA of TL106 was sequenced by Majorbio Bio-Pharm Biotechnology Co., Ltd., Beijing, China. For Pacific Biosciences sequencing, 8–10 k insert whole genome shotgun libraries were generated and sequenced on a Pacific Biosciences RS instrument using standard methods. An aliquot of 8 µg DNA was centrifuged in a Covaris g-TUBE (Covaris, MA, USA) at 6,000 rpm for 60 s using an Eppendorf 5424 centrifuge (Eppendorf, NY, USA). DNA fragments were purified, end-repaired and ligated with SMRTbell sequencing adapters following manufacturer’s recommendations (Pacific Biosciences, CA). Resulting sequencing libraries were purified three times using 0.45× volumes of Agencourt AMPure XP beads (Beckman Coulter Genomics, MA) following the manufacturer’s recommendations. Repeated sequences were removed leaving complete genome sequences.

### Genome annotation

The genes of nuclear genome were predicted using Glimmer 3.02 (http://www.cbcb.umd.edu/software/glimmer/) [25] and the genes of plasmid were carried out using GeneMark (http://topaz.gatech.edu/GeneMark/) [26]. Genes of tRNA and rRNA were analysed using tRNAscan-SE v1.3.1 and Barrnap 0.4.2 [27]. Repetitive sequences were predicted using RepeatMasker software (Version open-4.0.5) [28]. Tandem repeat sequences were analyzed using TRF (Tandem Repeats Finder) software [29]. Additionally, sequences of Genomic islands, CRISPR-Cas and Prophages were predicted using IslandViewer (http://www.pathogenomics.sfu.ca/islandviewer/browse/), CRISPRFinder (http://crispr.i2bc.paris-saclay.fr/) and PHAST (http://phast.wishartlab.com/index.html), respectively. Homology gene sequences and predicted protein sequences were analyzed in the NCBI database using Blastx or Blastp [30]. Predicted amino acid sequences of a protein were aligned with using the databases, including COG (Clusters of Orthologous Genes) (https://www.ncbi.nlm.nih.gov/COG/) [31] and KEGG (Kyoto Encyclopedia of Genes and Genomes) [32]. CAZyme genes were identified based on the CAZyme (carbohydrate active enzyme database) (http://www.cazy.org/) [33].

### Evolutionary analysis of strain TL106

Complete genome sequences of related taxa obtained from GenBank were annotated and translated into protein sequences using Prokka software (https://github.com/tseemann/prokka). Relationship of evolution among 20 *Bacillus* species was inferred using the maximum likelihood method and a phylogenetic tree was constructed by the Orthofinder (https://github.com/davidemms/OrthoFinder) [34]. Then, evolutionary tree was drawn using the online software ITOL (Interaction tree of life). A bootstrap analysis with 1000 replicates was conducted to obtain confidence levels for branches.

### Optimization of enzyme production conditions

The un-optimized medium contained soybean meal (0.99 g), corn flour (1.8 g), KH_2_PO_4_ (0.009 g), Na_2_HPO_4_ (0.12 g); The inoculation amount was 1.5% to the final fermentation volume. The un-optimized condition for fermentation was at 37 ℃ and at 220 rpm/min shaker speed for 24 h. To optimize the fermentation condition for cellulase production, single factor analysis was employed to compare the effects of different levels of soybean meal (0.39 g, 0.69 g, 0.99 g, 1.29 and 1.59 g), corn flour (1.2 g, 1.8 g, 2.4 g, 3.0 and 3.6 g), KH_2_PO_4_ (0.003 g, 0.006 g, 0.009 g, 0.012 and 0.015 g), Na_2_HPO_4_ (0.06 g, 0.12 g, 0.18 g, 0.24 and 0.30 g) on the production of cellulase in medium. We also evaluated different inoculation amounts (0.5%, 1.0%, 1.5%, 2.0% and 2.5%), fermentation temperatures (28℃, 31℃, 34℃, 37℃ and 40℃), fermentation times (0 h, 12 h, 24 h, 36 and 48 h), and shaker speeds (180 rpm, 200 rpm, 220 rpm, 240 rpm and 260 rpm) to optimize the fermentation condition. In each evaluation, cellulase activity in the fermentation supernatant was determined, and the optimal fermentation process of strain TL106 was determined based on cellulase activity. Activity of cellulase, amylase, xylanase and β-glucanase were determined using the DNS method [35].

One unit of cellulase activity was defined as the amount of enzyme required to produce 1 µmol of reducing sugar from a sodium carboxymethyl cellulose solution (4 mg/ml) per minute at 37 °C and pH 5.5. One unit of amylase activity was defined as the amount of enzyme required to yield 1 µmol of glucose from soluble starch per minute at 55 °C and pH 6.0. One unit of xylanase activity was defined as the amount of enzyme required to degrade 1 µmol of reducing sugar from xylan solution (5 mg/ml) per minute at 37 °C and pH 5.5. One unit of β-glucanase activity was defined as the amount of enzyme required to produce 1 µmol of glucose from β-glucan per minute at 55 °C and pH 5.6.

### Preparation of fermentation supernatant by fermenter

Based on the optimized condition of enzyme production, strain TL106 was cultured in the enzyme production medium containing soybean meal (0.69 g), corn flour (3.0 g), KH_2_PO_4_ (0.012 g) and Na_2_HPO_4_ (0.24 g). The inoculation amounts was 1.5%. Fermentation was performed at 34℃ for 12 h at the shaker speeds of 220 rpm. Then the bacteria solution of TL106 (200 mL) was inserted into the fermenter (BLBIO-30SJA-AUTO) containing the medium (11 L) for further cultivation (Culture conditions: fermentation temperature, 34℃; shaker speeds: 220 r/min; pH, 7.5; ventilation, 12 vvm; dissolved oxygen, 15–40%). The bacteria cultured medium after being fermented for 24 h in fermenter was centrifuged at 10,000 rpm at normal temperature for 10 min. The supernatant was fermented crude enzyme of TL106.

### Characterization of cellulose degradation activity

Crushed highland barley (100 g) and crushed wheat (100 g) were sterilized at 121.0℃ under the pressure of 103.4 kPa for 20 min which were loaded into bottles and sealed. The crude enzyme solution of strain TL106 (170 mL) was added into the experimental group and sterile distilled water (170 mL) was added into the control group. Followed by thoroughly mixed, bottles were incubated at 34 ± 2℃ for 48 h. After fermentation, highland barley and wheat samples were retrieved and dried at 105℃ to constant weights. The concentrations of crude fiber (CF), acid detergent fiber (ADF), neutral detergent fiber (NDF), starch, arabinoxylan and β-glucan in highland barley and wheat before and after fermentation were determined [36]. Degradation rates of CF, NDF, ADF, starch, arabinoxylan and β-glucan were calculated. Starch was determined using the starch content assay kit (Solarbio, Beijing, China), arabinoxylan was determined using the D-xylose assay kit (Megazyme Ireland, Wicklow, Ireland) and β-glucan was determined using the β-D-glucan assay kit (Megazyme Ireland, Wicklow, Ireland), respectively.

### Statistical analyses

All statistical analyses were evaluated by one-way analysis of variance using IBM SPSS statistics software. Duncan’s test was used to test for significant differences among treatments. Statistical differences were declared at *P <* 0.05. Mean values and standard deviations of triplicate measurements were calculated to plot the graphs and histograms.

## Results

### Genome features and annotation

**Fig. 1:**
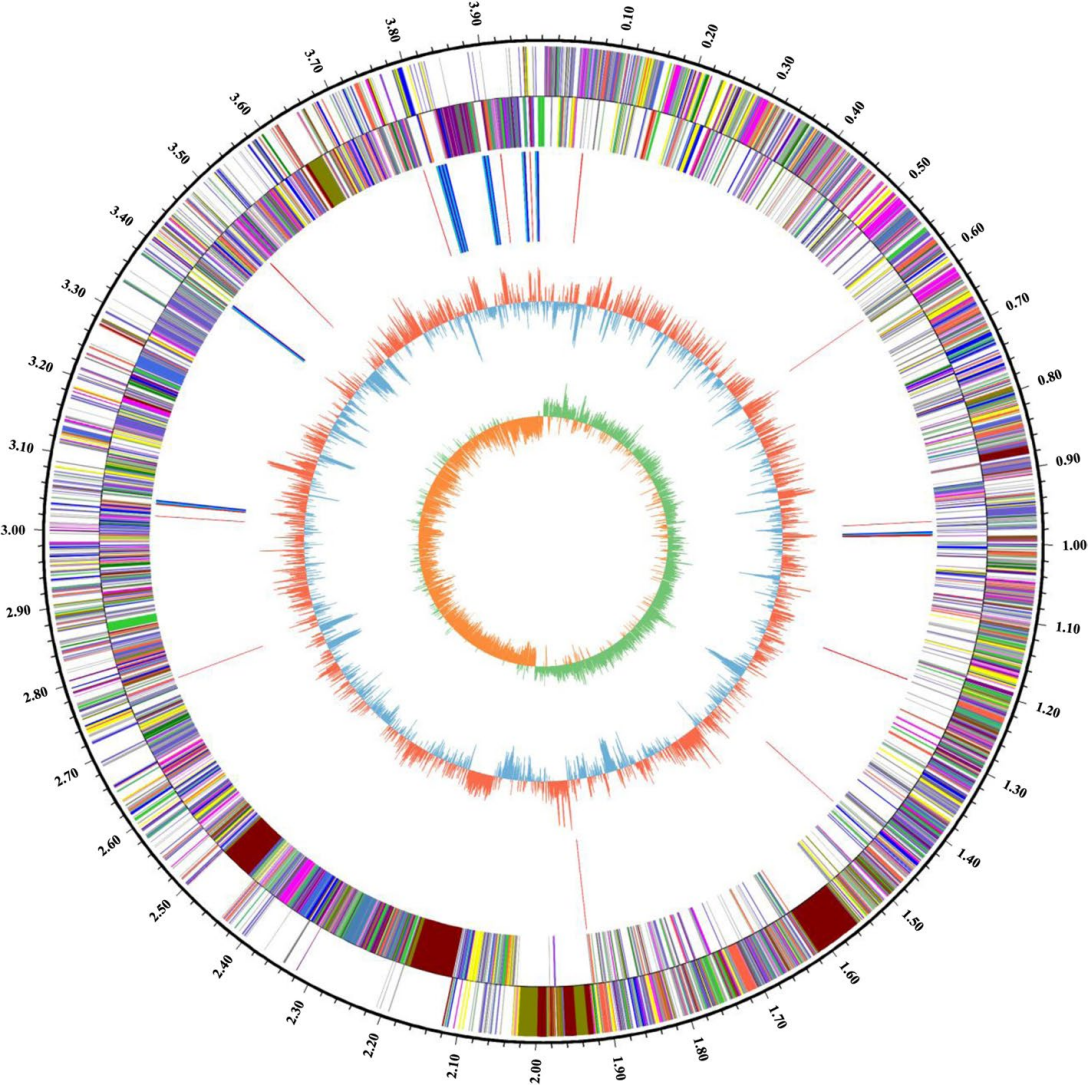
Circular graph of TL106. The outermost circle is the physical coordinates of genome sequence. From the outer to the innermost circle, depicts the ORFs on the positive, the ORFs on the negative strands (different colors indicate different COG functions), tRNA and rRNA, GC content (red or blue represent the GC content higher or lower than average of GC content) and GC-skew (green > 0, yellow < 0), respectively

Sequencing and de novo assembly results showed that the genome length of TL106 was 3,997,876 bp and the GC content (the percentage of Guanine and Cytosine in genome) was 46.54%. The complete genomes of strain TL106 consisted of one chromosome (3,980,960 bp) and one plasmid (16,916 bp). The whole genomes encode a total of 4,130 genes, including 23 tRNA genes and 16 rRNA genes. Of these genes, 3,016 were predicted functions, 1,114 were unknown functions. In addition, 2 Prophages, 6 CRISPR-Cas, 278 Promotor and 4 Genomic islands were found in the whole genomes. This whole genome sequence has been deposited at GenBank under the accession no. PRJNA787390. The strain TL106 genomic features of TL106 were summarized in Supplementary Table S1 and graphically represented in Fig. 1.

### COG classification and KEGG analysis

**Fig. 2:**
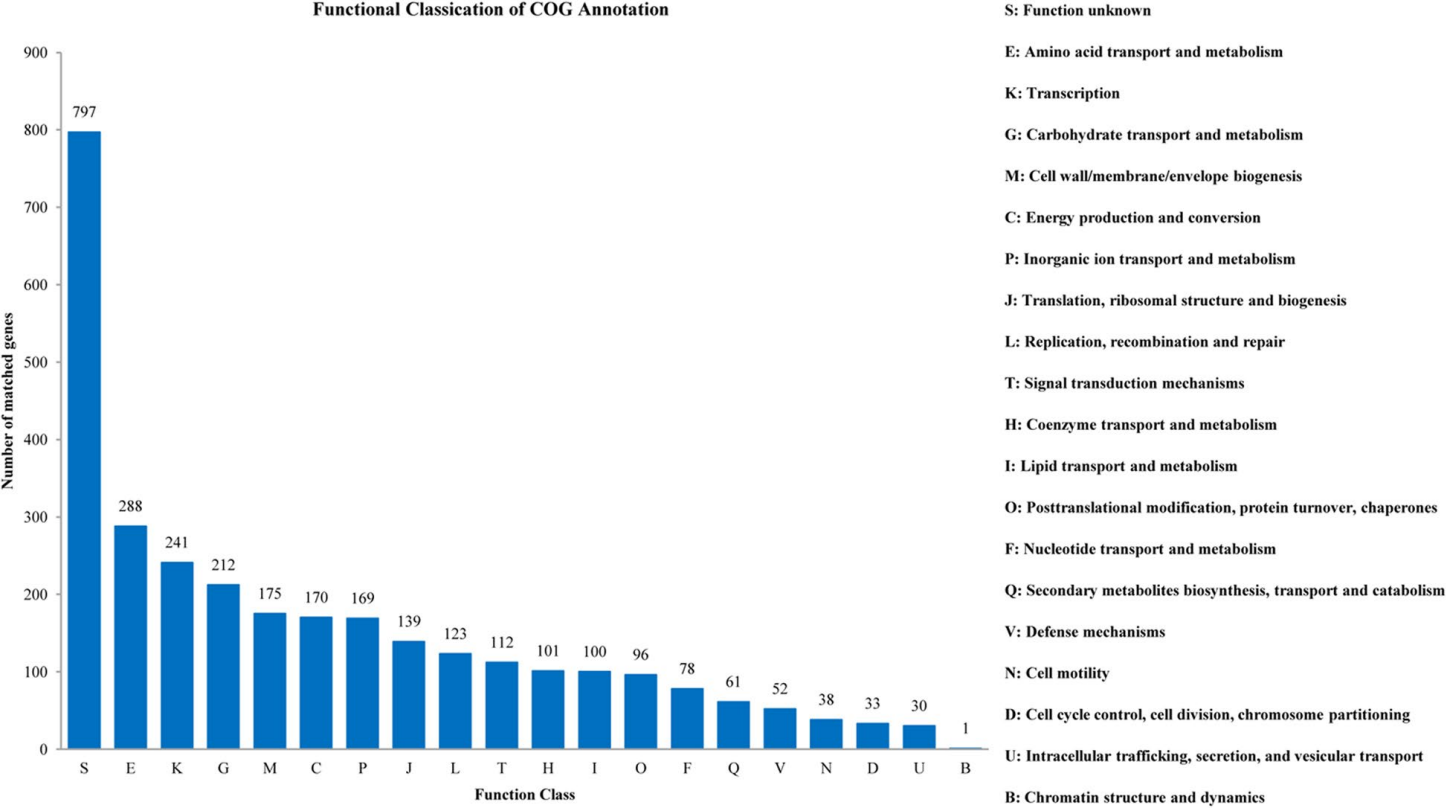
COG classification for the strain TL106 genes

A high proportion of genes (73.02%) were annotated in COG. Classified genes (3,016) were obtained in 20 COG functional categories (Fig. 2), which included five main functional groups: function unknown (797 genes), amino acid transport and metabolism (288 genes), transcription (241 genes), carbohydrate transport and metabolism (212 genes) and cell wall/membrane/envelope biogenesis (175 genes). The least represented groups included defense mechanisms (52 genes), cell motility (38 genes), cell cycle control, cell division, chromosome partitioning (33 genes), intracellular trafficking, secretion, and vesicular transport (30 genes), chromatin structure and dynamics (1 gene).

**Fig. 3:**
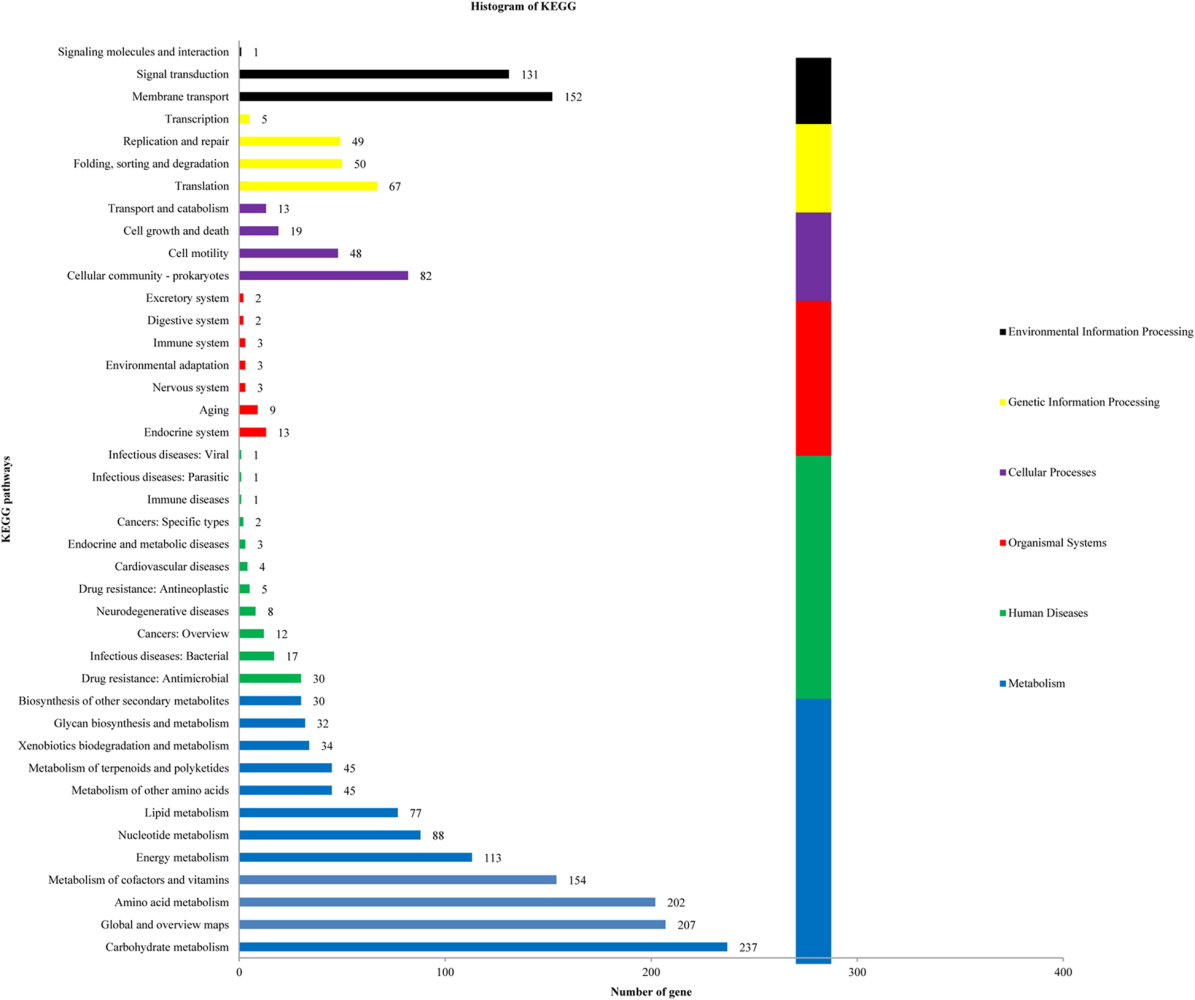
KEGG pathway analysis for the strain TL106 genes

Genes (2,000) were categorized into 6 big classes and 41 subclasses based on KEGG categorization (Fig. 3), involved cellular processes, metabolism, human diseases, genetic information processing, organismal systems, and environmental information processing. The 41 subclasses included the genes involved carbohydrate metabolism (237 genes), global and overview maps (207 genes), amino acid metabolism (202 genes), metabolism of cofactors and vitamins (154 genes), membrane transport (152 genes), signal transduction (131 genes), and energy metabolism (113 genes).

### CAZyme annotation

**Fig. 4:**
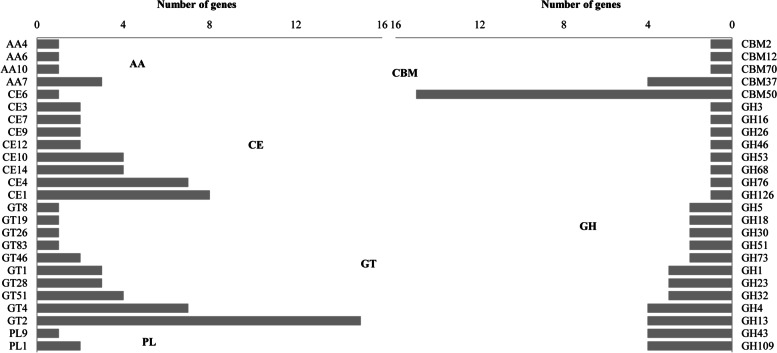
Annotation of the carbohydrate active enzyme of TL106. GH represents glucoside hydrolase; GT represents glycosyl transferase; CE represents carbohydrate esterase; PL represents polysaccharide lyase; AA represents auxiliary activities; and CBM represents carbohydrate binding modules

A total of 144 CAZyme genes were annotated in TL106, accounting for 3.48% of all encoding genes (Fig. 4). Among those genes, glucoside hydrolase (GH) plays a key role in carbohydrate degradation, and 43 GH genes were annotated in TL106 genome. These 43 genes were grouped into 20 different GH families. Besides, 38 glycosyl transferase (GT) genes, 32 carbohydrate esterase (CE) genes, 3 polysaccharide lyase (PL) genes, 6 auxiliary activities (AA) genes and 22 carbohydrate-binding modules (CBM) genes were identified in TL106. Ten GT families accounted for 38 GT genes and GT2 had the largest number of genes. Comparative genome analysis among TL106 and other different *B. amyloliquefaciens*, including *B. Amyloliquefaciens* HK1, *B. amyloliquefaciens* EA19, *B. amyloliquefaciens* LL3 and *B. amyloliquefaciens* XH7, clearly showed that they shared different distribution patterns of CAZyme genes. Specifically, the CE and AA families of TL106 contained more CAZyme genes than the other four strains (Supplementary Table S2). The result of CAZyme annotation indicated that TL106 may contain more types of enzyme active in degrading carbohydrat.

### Analysis of cellulase and hemicellulase genes in TL106

There were 4 β-glucosidase genes and 1 endoglucanase gene detected in TL106 with NR database (NCBI non-redundant protein sequences), β-glucosidase can degrade cellobiose and cellulose dextrin into glucose. Endoglucanase can severe β-1,4 glycosidic bonds and hydrolyze cellulose to oligos. Both the β-glucosidase and endoglucanase genes are cellulase genes. The proteins encoded by β-glucosidase and endoglucanase genes belong to GH1, GH73 and GH5, respectively. In addition, 11 hemicellulose genes, such as the genes of ß-glucanase, xylanase, glucuronoxylanase, ß-mannosidase, ß-galactosidase, α-arabinofuranosidase, endo-α-L-arabinosidase, and arabinofuranohydrolase were annotated in TL106. The protein encoded by hemicellulose genes belongs to GH16, GH11, GH30, GH1, GH53, GH51, GH43 and GH26 (Table 1). The results of annotation revealed that TL106 had the potential to degrade cellulose.

**Table 1:** Cellulase and hemicellulase genes in the genome of strain TL106

Enzyme classification	Gene ID^a^	CAZy modules	Enzyme classification	Gene ID^a^	CAZy modules
ß-glucosidase	gene0240	GH1	ß-galactosidase	gene2807	GH1
	gene0256	GH1		gene2813	GH53
	gene3855	GH1	α-arabinofuranosidase	gene1321	GH51
	gene0562	GH73		gene1343	GH51
Endoglucanase	gene2152	GH5	Endo-α-L-arabinosidase	gene0176	GH43
ß-glucanase	gene0204	GH16		gene1312	GH43
Xylanase	gene4042	GH11	Arabinofuranohydrolase	gene2147	GH43
Glucuronoxylanase	gene2148	GH30	ß-mannosidase	gene0237	GH26

^a^Gene ID: In this study, 4130 genes were obtained from the whole genome of strain TL106. Each gene was identified as “Gene + Number” where number ranged from 1 to 4130

### Evolutionary analysis of strain TL106

**Fig. 5:**
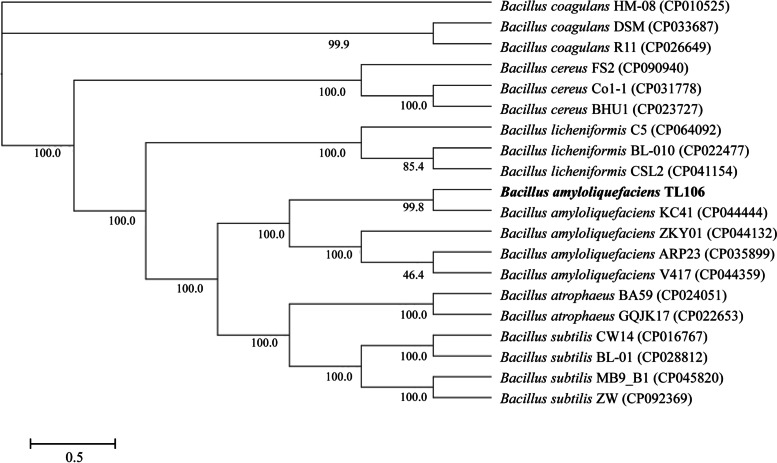
Phylogenetic tree reconstructed based on the complete genome sequence to analyze the relationship between TL106 and its related bacterial species. The numbers in parentheses represent the sequence accession numbers in GenBank

The relationship between TL106 and its related bacterial species was analyzed by phylogenetic tree (Fig. 5). The analyses based upon the complete genome sequence of strain TL106. It showed that the sequence of TL106 was formed a cluster with *B. amyloliquefaciens* KC41, ZKY01, ARP23 and V417. Therefore, the strain used in this experiment was identified as *B. amyloliquefaciens*, and named *B. amyloliquefaciens* TL106.

### Optimization of enzyme production conditions

Cellulase activity of strain TL106 reached the maximum (7.70 U/mL and 10.48 U/mL) when the content of soybean meal and corn flour in enzyme producing medium were 0.69 and 3.0 g, respectively (Supplementary Fig. S1, A and B). Cellulase activity increased with the contents of KH_2_PO_4_ (0.012 g) and Na_2_HPO_4_ (0.24 g) reached. Contents of KH_2_PO_4_ and Na_2_HPO_4_ higher than 0.012 and 0.24 g decreased the cellulase activity in the culture medium (Supplementary Fig. S1, C and D). The cellulase activity reached highest when the fermentation lasted for 12 h at 34℃. After these peaks, cellulase activity decreased with prolonged fermentation time and fermentation temperature (Supplementary Fig. S1, E and F). Similarly, cellulase activity showed peak when the inoculation amount was 1.5% with the shaking speed at 220 rpm (Supplementary Fig. S1, G and H). In summary, the optimized conditions for the fermentation of strain TL106 were achieved with 0.69 g soybean meal, 3.0 g corn flour, 0.012 g KH_2_PO_4_, 0.24 g Na_2_HPO_4_, at the fermentation temperature of 34℃ for 12 h with the inoculation amount of 1.5% at the shaking speed of 220 rpm.

**Fig. 6:**
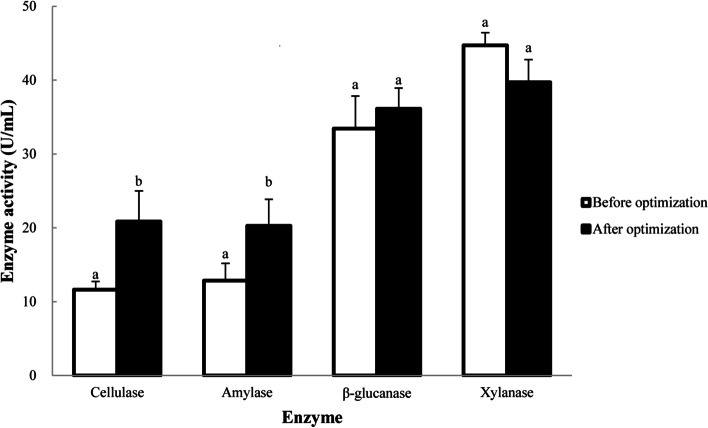
The activities of cellulase, amylase, β-glucanase, xylanase in the TL106 before and after optimization of enzyme production conditions

The activities of cellulase, amylase, β-glucanase and xylanase were determined before and after the optimization of fermentation conditions. The activities of cellulase and amylase were increased (*P < 0.05*) after the optimization, in which cellulase activity was increased from 11.61 U/mL to 20.86 U/mL and amylase activity was increased from 12.84 U/mL to 20.25 U/mL. However, β-glucanase and xylanase activities did not change (*P *> *0.05*; Fig. 6).

### Degradation characteristics of strain TL106

**Fig. 7:**
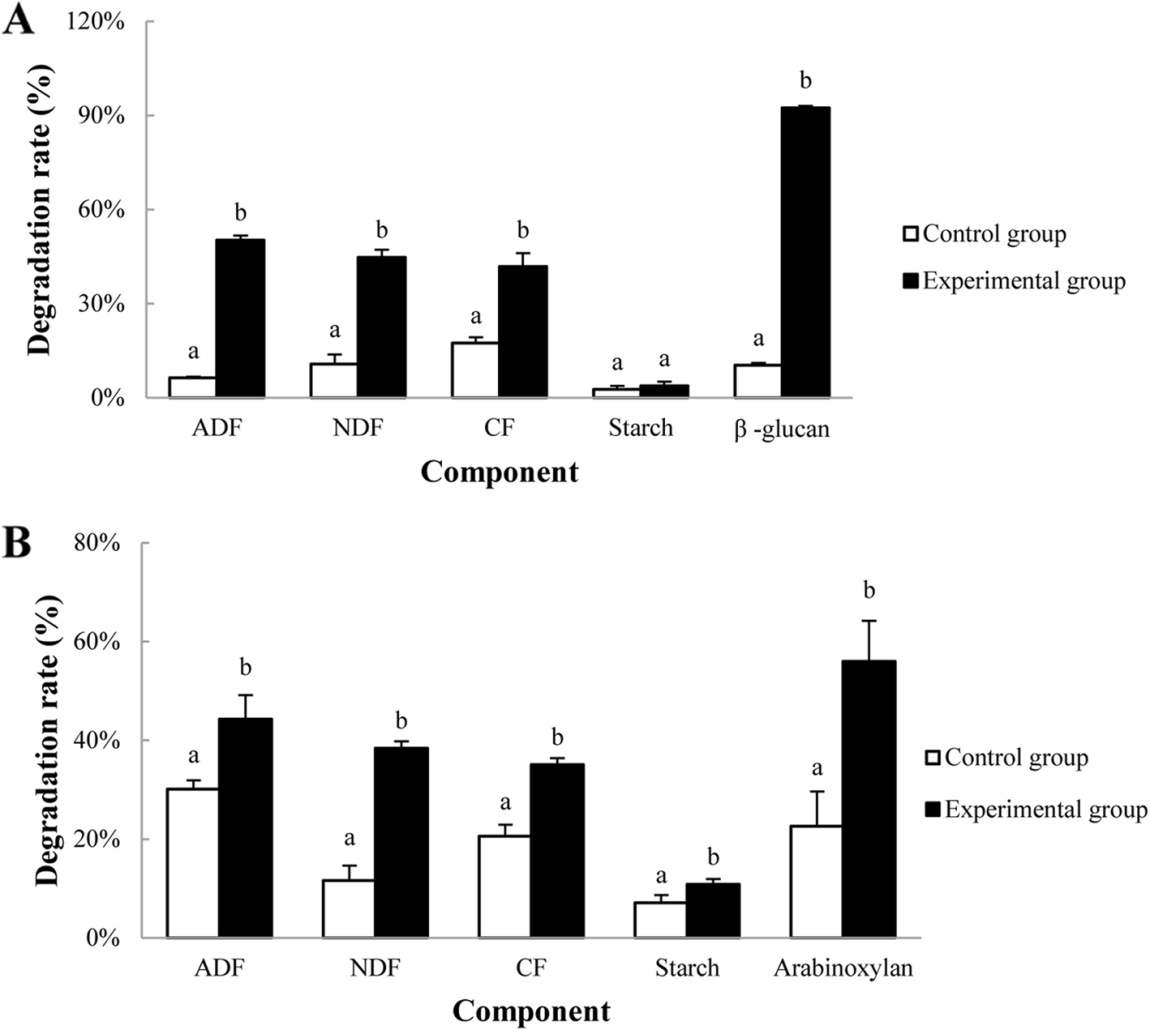
Degradation rate of various substances in highland barley and wheat. **A** Degradation rate of ADF, NDF, CF, starch and β-glucan in highland barley; **B** Degradation rate of ADF, NDF, CF, starch and arabinoxylans in wheat

In highland barley (Fig. 7A), the degradation rate of acid detergent fiber, neutral detergent fiber, crude fiber and β-glucan in the experimental group were increased (*P *< *0.05*) compared with the control group, especially degradation of β-glucan, which increased nearly 9 times. Starch degradation was unaffected. The bacterial solution of strain TL106 significantly increased the degradation of acid detergent fiber, neutral detergent fiber, crude fiber, starch and arabinoxylan in wheat (Fig. 7B). Degradation of arabinoxylan was more than doubled. All the above results indicated that strain TL106 had the ability to degrade cellulose.

## Discussion

Recently, some high-fiber feedstuffs, such as wheat bran, rapeseed meal, and cottonseed meal, have been increasingly used in feed manufacture for monogastric animals. However, due to their high fiber contents, these ingredients are difficult for monogastric animals to digest. The low digestibility leads to excessive waste of limited feed resources [37]. Generally, cellulase, the produced via fermentation of bacteria or fungi possesses low activities in degradation of cellulose [38], which significantly limits its application in the feed industry. Therefore, it is important to exploit a new source of cellulase. *B. amyloliquefaciens* is a cellulose-degrading bacterium that is rich in CAZymes genes and can produce a variety of cellulose-degrading enzymes [39]. A full understanding of the *B. amyloliquefaciens* genome will aid exploitation of cellulolytic enzymes produced by this organism. In this study, a new *B. amyloliquefaciens* strain, *B. amyloliquefaciens* TL106, isolated from Tibetan pig feces was idenified. It can produce cellulase with high performance. We studied the cellulose degrading potential of this organism using genome sequencing analysis and in vitro experiments. Genome sequence analysis indicated that total gene length of strain TL106 was 3,997,876 bp, and the content of GC was 46.54%, which was similar to *B. amyloliquefaciens* TF28 [40].

COG function annotation revealed that many gene coding products can participate in amino acid transport and metabolism, carbohydrate transport and metabolism. However, a total 1114 genes in TL106 have not been functionally annotated in COG, so that many genes need to be further explored. KEGG function annotation revealed that about 11.85% and 10.10% of predicted proteins in TL106 may regulate carbohydrate metabolism and amino acid metabolism, respectively. These proteins will greatly help strain TL106 establish a powerful metabolic regulation system which is adaptable to different living environments and may assist in regulating physiological and biochemical functions in animals [41]. In this research, evolutionary relationships between TL106 and other *Bacillus* species were evaluated based on the whole genome sequence. The whole genome sequence of TL106 and *B. amyloliquefaciens* KC41, ZKY01, ARP23 and V417 were clustered together by evolutionary tree, both for *B. amyloliquefaciens*.

CAZymes played a crucial role in breaking down carbohydrates such as cellulose, proteoglycans and starch into mono or oligo saccharides which can be absorbed by intestinal epithelia. Most CAZymes in intestines were encoded by gut microbiota [9]. Varying CAZyme profiles of intestinal microbes may had different capabilities of carbohydrate metabolism [42]. Comparison of CAZyme genes in five *B. amyloliquefaciens* strains, including TL106, HK1, EA19, LL3 and XH7 revealed that *B. amyloliquefaciens* TL106 had more types of CAZymes genes than the other four strains [43–46]. GH genes were took a large part in the genome of *B. amyloliquefaciens*, consistent with those of the genome of *Aspergillus fumigatus* Z5, which showed highly efficient cellulose-degradation capability [47]. *B. amyloliquefaciens* TL106 contained 144 genes encoding CAZyme, which belong to GH, GT, CE, PL and AA families. The GH family enzymes can hydrolyze glycosidic bonds [48]. There were 20 GH family members in strain TL106, of which GH1, GH3, GH5 and GH73 encoded cellulase, and GH5 mainly encoded endoglucanase. GH1, GH3 and GH73 were primarily β-glucosidases [49, 50]. They could coordinately degrade cellulose. CAZymes that can degrade hemicellulose were also identified in strain TL106, such as GH26, GH32, GH43, GH51 and GH53. Among them, GH26 predominantly comprises β-1,4 mannanases, which can hydrolyze mannan, galactomannan and glucomannan [51]. GH32 family enzymes, can hydrolyze or synthesize of fructosan glycoside bonds, including fructanase and sucrase [52]. GH43 was an important member of xylan degradation [53]. GH51 can dissociate arabinogalactan and arabinomannan in cell walls, promoting the degradation of pectin [54]. GH53 had been reported as an endo-1,4-β-galactanase and had the ability to hydrolyze 1,4-β-D-galactoside bonds [55]. Carbohydrate esterases related to xylan decomposition were identified in strain TL106, including CE3, CE4, CE7 and CE10. CE3, CE4 and CE7 were acetyl xylan esterases which could promote dissolution of xylan [56]. CE 10 was carboxylesterase and xylanase to degrade hemicellulose [57]. *B. amyloliquefaciens* TL106 also contains AA4, a vanillyl alcohol oxidase which could transform certain phenols [58], and AA7 that transform lignocellulosic to fermentable momosaccharides [59]. We also found that TL106 contained CBM2 [60]. The CBM2 family enzymes contained members that bind cellulose (CBM2a) and xylan (CBM2b). *B. amyloliquefaciens* TL106 also included multiple cellulase genes and hemicellulase genes, such as ß-glucosidase genes, endoglucanase genes, ß-glucanase genes and xylanase genes. Abundant cellulase and hemicellulase genes also indicated that strain TL106 has the potential of degrading cellulose.

Enzyme production by microbial fermentation is strongly associated to a variety of factors, including the amounts of carbon source, nitrogen source, inorganic salts in the enzyme production medium, fermentation temperature, fermentation time, shaking speed, and inoculation amount. It is necessary to optimize the fermentation conditions to maximize the production from targeted bacteria [61]. In this study, single factor method was used to optimize conditions for fermentation of strain TL106. Compared with the initial fermentation conditions, cellulase activity of crude enzyme solution after optimization increased 79.67%, to 20.86 U/mL. This result was consistent with that of reported *B. amyloliquefaciens* MBAA3 [62].

Cellulase is a highly active biocatalyst which can degrade cellulose, and cellulase activity is the key factor for efficient utilization of cellulose [63]. Currently, many cellulase producing strains have low enzyme activity [64]. How to improve cellulase activity and reduce the production cost is the important technical issue for the application of cellulase in industry. Ma et al. [65] reported that the maximum cellulase activity produced by *B. subtilis* BY-4 isolated from the gut of Tibetan pig was 8.61 U/mL. Meng et al. [66] found that *B. subtilis* BY-3 exhibited maximum cellulase activity of up to 4.32 U/mL. The cellulase activity of strain TL106 was higher than BY4 or BY3, which suggested that *B. amyloliquefaciens* TL106 had greater potential value for commercial application. Interestingly, the activities of amylase and β-glucanase in crude enzyme solution after optimization increased by 57.71% and 8.07% in fermented TL106, respectively. Amylase can hydrolyze α-1,4-glucan [67], and β-glucanase can hydrolyze β-glucan in wheat and barley [68]. These two enzymes are used widely in food industries, animal production, and biopharmaceutical ventures. So, TL106 may have broader application for enzyme production in the future. However, the activity of xylanase in crude enzyme solution of TL106 after optimization was decreased. This could be due to the purpose of optimization of fermentation conditions which was based on cellulase activity. The optimum conditions for cellulase production may not be suitable for that of xylanase.

A high-fiber diet can reduce digestibility of nutrients, because of increased chyme emptying rate, short residence time of chyme in the gastrointestinal tract, reduced contact of digestive enzymes with feed and chyme nutrients in the intestine [69]. Dietary fiber also affects the net energy value in diets [70]. Magistrelli et al. (2009) [71] reported that feeding high-fiber diets to growing-finishing pigs markedly reduced the average daily gain, loin muscle area, and leg muscle weight. Wang et al. [72] reported average daily gain (ADG) and average daily feed intake (ADFI) of piglets were negatively correlated with dietary fiber level. Therefore, improvement of degradation of fiber components in feed is an essential issue for increasing digestibility of feed. In this study, the degradation rates via fermentation by the crude enzyme of strain TL106 of crude fiber, acid detergent fiber, and neutral detergent fiber in wheat and hulless barley were 35.09% and 41.79%, 38.39% and 44.72%, 44.28% and 50.26%, respectively. Besides efficient degradation of fibers, the fermentation solution of strain TL106 could degradate wheat and barely, which were composed of arabinoxylan and β-glucan [73]. As anti-nutritional factors, arabinoxylan and β-glucan widely existed in grains feed, such as wheat, oats, rye and barely, are characterized by high viscosity, high hydrophobicity and high ion adsorption, which can increase the viscosity of chyme in animal intestines. Increased viscosity of chyme limits efficiency of digestive enzymes on digestibility of nutrients in feed [74, 75]. In feed production, xylanase and β-glucanase that can degrade arabinoxylan and β-glucan in feed are highly needed [76]. Strain TL106 could produce xylanase and β-glucanase which was vital characteristic for its potential of wide application in feed industry.

In conclusion, the genome of the *B. amyloliquefaciens* TL106 was sequenced and analyzed in the present study. A total of 144 genes were predicted to be related to carbohydrate active enzyme, including GH, GT, CE, PL, AA and CBM, which indicated that strain TL106 contained many types of carbohydrate hydrolases. The maximum cellulase activity in crude enzyme solution of TL106 was 20.86 U/mL after optimation of fermentation conditions, higher than other *Bacillus*. Besides, TL106 could produce amylase, β-glucanase and xylanase. TL106 could not merely improve the degradation rate of fiber, but also the degradation rate of anti-nutritional factors (arabinoxylan and β-glucan). Therefore, strain TL106 had great potential being applied in feed enzyme production for improvement of using high fiber contained grain feed.

## Supplementary Information

**Additional file 1.**

**Additional file 2.**

## Data Availability

All data generated or analyzed during this study are included in this published article. The datasets presented in this study can be found in online repositories. The names of the repository/repositories can be found at: https://www.ncbi.nlm.nih.gov/, the accession number is PRJNA787390.
