# Supplementary material for: Complete genome sequencing and investigation on the fiber-degrading potential of Bacillus amyloliquefaciens strain TL106 from the tibetan pig
Source: BMC Microbiol. 2022 Jul 29;22:186. doi: 10.1186/s12866-022-02599-7 (PMC9336001; doi:10.1186/s12866-022-02599-7)
Supplement: Supplementary file 1 — Additional file 1. [file 12866_2022_2599_MOESM1_ESM.docx]

**Complete genome sequencing and investigation on the fiber-degrading potential of *Bacillus amyloliquefaciens* strain TL106 from the Tibetan pig**

Zhenda Shang^1,2^, Suozhu Liu^2^, Yanzhen Duan^2^, Chengling Bao^1^, Jian Wang^1^, Bing Dong^1^, and Yunhe Cao^1*^

1 State Key Laboratory of Animal Nutrition, College of Animal Science and Technology, China Agricultural University, Beijing 100193, People's Republic of China

2 College of Animal Science, Tibet Agricultural and Animal Husbandry University, Nyingchi 860000, People's Republic of China

*Corresponding author at: College of Animal Science and Technology, China Agricultural University, Beijing, 100193, People's Republic of China.

E-mail: [shangzhenda1988@163.com](mailto:shangzhenda1988@163.com) (Z.D. Shang), [caoyh@cau.edu.cn](mailto:caoyh@cau.edu.cn) (Y.H. Cao).

**Supplementary Table S1.** Genome statistics of TL106

| Gene number | 4130 |
| --- | --- |
| Gene total length (bp) | 3997876 |
| GC/% | 46.54 |
| Gene density | 1.03 genes per kb |
| GC content in gene region (%) | 47.34 |
| Gene/genome (%) | 88.88 |
| Intergenetic region length (bp) | 444421 |
| GC content in intergenetic region (%) | 40.14 |
| Intergenetic length/genome (%) | 11.12 |

**Supplementary Table S2.** CAZyme gene numbers in *B. amyloliquefaciens* genomes

| Species of *B. amyloliquefaciens* | CAZyme proteins | GH | GT | CE | PL | AA | CBM |
| --- | --- | --- | --- | --- | --- | --- | --- |
| TL106 | 144 | 43 | 38 | 32 | 3 | 6 | 22 |
| HK1 | 118 | 45 | 34 | 8 | 3 | 1 | 27 |
| EA19 | 102 | 38 | 27 | 7 | 3 | 1 | 26 |
| LL3 | 115 | 45 | 36 | 8 | 3 | 1 | 22 |
| XH7 | 114 | 44 | 35 | 8 | 3 | 1 | 23 |

Genomes: HK1 (GenBank: CP018902.1) (Zhang *et al*. 2018); EA19 (GenBank: CP079834.1) (Zeng *et al*. 2021); LL3 (GenBank: CP002634.1) (Geng *et al*. 2011); XH7 (GenBank: CP002927.1) (Yang *et al*. 2011).

GH: Glucoside hydrolase; GT: Glycosyl transferase; CE: Carbohydrate esterase; PL: Polysaccharide lyase; AA: Auxiliary activities; CBM: Carbohydrate binding modules.


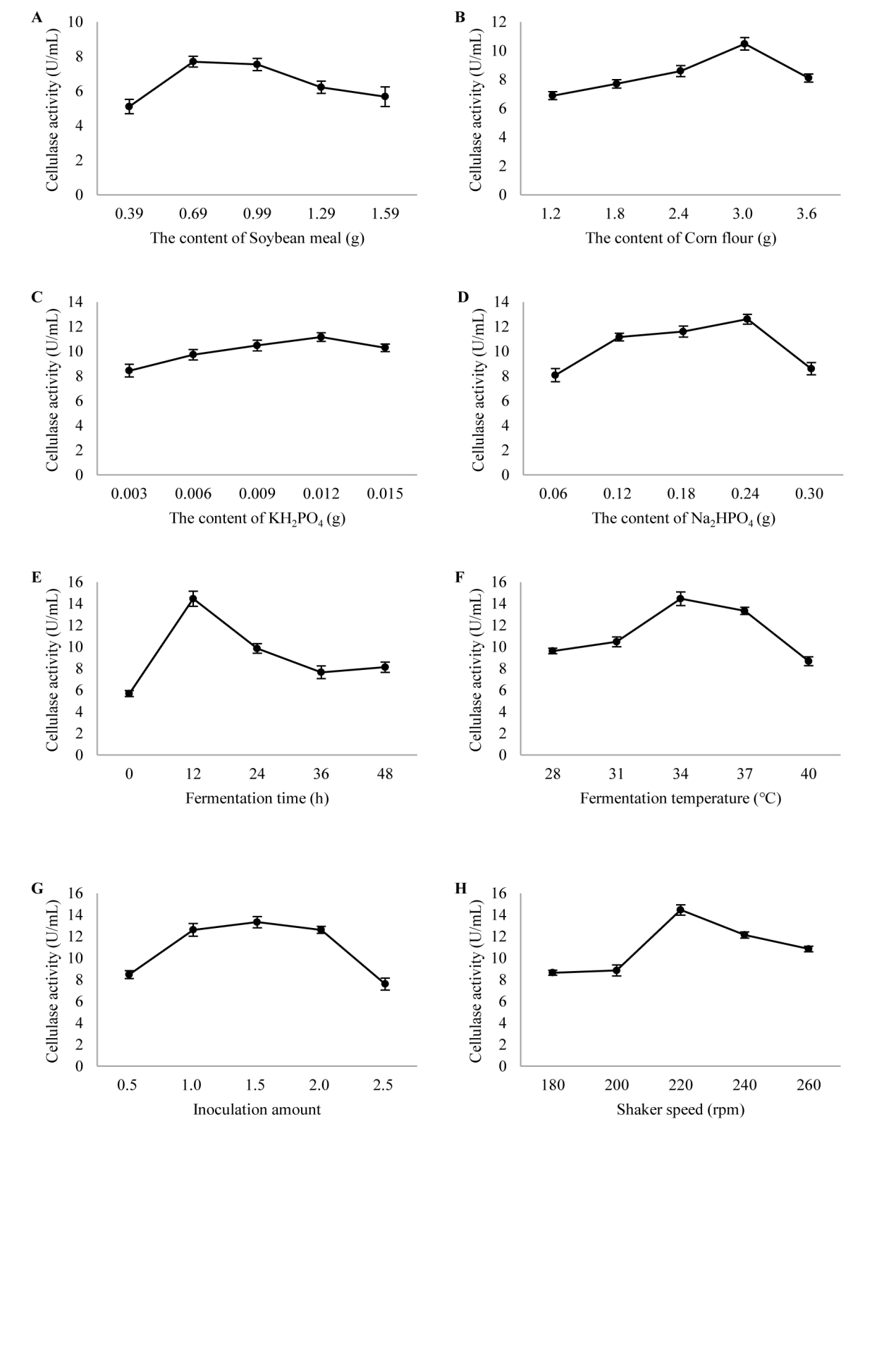


**Supplementary Fig. S1** Effect of enzyme producing medium composition and fermentation conditions on cellulase production. A: Effect of the content of soybean meal on cellulase production; B: Effect of the content of corn flour on cellulase production; C: Effect of the content of KH2PO4 on cellulase production; D: Effect of the content of Na2HPO4 on cellulase production; E: Effect of the inoculation amount on cellulase production; F: Effect of the fermentation temperature on cellulase production; G: Effect of the fermentation time on cellulase production; H: Effect of the shaker speed on cellulase production.
